# THE USE OF SURGICAL ADHESIVE AND SUTURE FIXING MESHES TO THE ABDOMINAL WALL: AN EXPERIMENTAL STUDY IN RATS

**DOI:** 10.1590/0102-672020210002e1649

**Published:** 2022-06-17

**Authors:** Carlos Alberto Lima UTRABO, Cesar Roberto BUSATO, Mário Rodrigues MONTEMÓR-NETTO, Leandro Cavalcanti LIPINSKI, Vitória Rossetim CELINSKI, Mylena Fernanda FERRONATO, Osvaldo MALAFAIA, Adriana Yuriko KOGA

**Affiliations:** 1 School of Medicine, State University of Ponta Grossa, Ponta Grossa, PR, Brazil;; 2 Evangelical Mackenzie Faculty of Paraná, Curitiba, PR, Brazil;; 3 Federal University of Paraná, Curitiba, PR, Brazil.

**Keywords:** Hernia, Abdominal wall, Surgical glue, Surgical meshes, Hérnia, Parede abdominal, Cola cirúrgica, Telas cirúrgicas

## Abstract

Proper fixation of the surgical mesh determines the success of a herniorrhaphy. Understanding the inflammatory response and the mechanical properties of the mesh helps to define whether a fixation method is superior.

**AIM:**

This study aimed to evaluate the healing of defects in the abdominal wall of rats, comparing the repair of macroporous polypropylene meshes fixed with surgical glue and polypropylene thread.

**METHODS:**

In 20 Wistar rats, a defect was produced in the abdominal wall, with the integrity of the parietal peritoneum. For correction, the meshes were fixed with surgical glue (2-octyl cyanoacrylate) (subgroup C1), or polypropylene suture (subgroup C2). The two subgroups of 10 animals were euthanized on the 90th postoperative day, and the fragments of the abdominal wall were submitted to macroscopic, histological, and tensiometric analysis.

**RESULTS:**

Macroscopic analysis did not show any abnormalities. Tensiometry on the 90th postoperative day in subgroup C1 showed mean rupture tension of 28.47N and in subgroup C2 32.06N (p=0.773). The inflammatory process score revealed that both groups are in the subacute phase (p=0.380).

**CONCLUSION:**

The fixation of a polypropylene macroporous mesh to repair an abdominal wall defect can be performed with surgical glue (2-octyl cyanoacrylate) or polypropylene suture, both methods being equally effective.

## RESUMO - RACIONAL

A adequada fixação da tela cirúrgica determina o sucesso de uma herniorrafia. Entender a resposta inflamatória e as propriedades mecânicas da tela contribui para definir se há superioridade de um método de fixação.

**OBJETIVO:**

Avaliar a cicatrização de defeitos em parede abdominal de ratos, comparando-se o reparo das telas de polipropileno macroporosas fixadas com cola cirúrgica e fio de polipropileno.

**MÉTODOS:**

Foi produzido defeito na parede abdominal de 20 ratos Wistar com integridade do peritônio parietal. Na correção, as telas foram fixadas com cola cirúrgica 2-octil cianoacrilato (C1) ou sutura (C2). Os dois subgrupos de 10 animais foram eutanasiados no 90º dia de pós-operatório e os fragmentos da parede abdominal foram submetidos a análise macroscópica, histológica e tensiométrica.

**RESULTADOS:**

A análise macroscópica não mostrou qualquer anormalidade. A tensiometria no 90º dia de pós-operatório no subgrupo C1 demonstrou tensão média de ruptura de 28,47N e no subgrupo C2 de 32,06N (p=0,773). O escore de processo inflamatório revelou que ambos os grupos se encontram na fase subaguda (p=0,380).

**CONCLUSÃO:**

A fixação de tela macroporosa de polipropileno para reparo de defeito em parede abdominal pode ser realizada com cola cirúrgica (2-octil cianoacrilato) ou sutura de polipropileno, sendo ambos os métodos igualmente eficazes.

## INTRODUCTION

Herniorrhaphy is one of the most performed surgical procedures worldwide^3^ ^,^ ^11^ ^,^ ^14^ ^,^ ^24^. The majority of these corrections are made with the use of surgical meshes^5^ ^,^ ^8^ ^,^ ^9^; this idea is grounded on the tension-free technique, which was proposed by Lichtenstein^7^. This strategy is recognized because it had a great impact on reducing the recurrence rates^4^ ^,^ ^15^ ^,^ ^18^.

In the beginning, meshes were traumatically fixed in the abdominal, which was made with sutures^6^ ^,^ ^10^. However, it was observed that patients submitted to this fixation technique presented a high incidence of chronic pain after the herniorhaphy^12^. It can be explained by the compression of the nerves and also by the aggression of the tissue that results from the permanence of the suture^2^ ^,^ ^17^ ^,^ ^21^.

Then, studies that are related to the use of surgical in the surgery correction of hernias attempted to find the ideal model ^1^ ^,^ ^7^ ^,^ ^23^. In this way, less invasive fixation methods were developed, which can be demonstrated by the advent of surgical glue^5^ ^,^ ^11^ ^,^ ^15^.

It was reported that the atraumatic method did not increase the incidence of recurrence and also was able to reduce chronic pain after herniorrhaphy procedure^12^ ^,^ ^15^ ^,^ ^18^ ^,^ ^20^. Furthermore, the adhesive was superior to suture in technical features such as low duration procedure, hematoma incidence, and recovery time to daily activities^2^ ^,^ ^21^.

Surgical glue has been presented as a secure alternative^12^. This resource has performed a great acceptability between surgeons because it is easy to use and also has satisfactory expression in the postoperative patients^6^. Although the results related to its use on the fixation of surgical meshes are limited, the potential after-effects have not been explored ^5^ ^,^ ^15^ ^,^ ^18^.

It is considered that the abdominal wall is a dynamic system that has to resist high pressures, so it is essential to preserve the flexibility of the wall after its correction with surgical mesh. To achieve this purpose, an ideal congruence between the implanted material and the abdominal muscles must be done^7^.

Moreover, it is known that the abdominal wall does not show a uniform behavior ^7^. This implies that many parameters must be considered in the final result of an intervention, such as the selection of a proper surgical technique and the individuality of each patient^25^.

When a surgical mesh is implanted in the abdominal, it results in inflammatory cells migration, such as neutrophils and macrophages, and in cytokines and growth factors^16^. In this way, chronic inflammation is a possible complication that can be related, and it is triggered by foreign body inflammatory reactions ^13^. It results in the loss of congruence with the tissue, in its contraction, and also in regional physicochemical properties^25^.

Therefore, the mechanical properties analysis of the mesh, its elasticity, and inflammatory response are necessary to determine the success of a surgical procedure^3^ ^,^ ^4^.

The collagen is the main element of extracellular matrix and helps to maintain the tension and elasticity of the tissue. Type I collagen is hard and widely distributed through the human body; it can be found in the fascia, skin, ligaments, and fibrous connective tissue, which are responsible for the mechanism of the tissues. Type III collagen appears at the beginning of the healing process and is less resistant. During the healing process, type III collagen is replaced by type I collagen, which is highly resistant. Therefore, the relations between types I and III collagen have a great influence on many parameters of the healing process^19^.

The present experiment aimed to evaluate the healing process in the closure of the abdominal wall of rats, comparing the application of polypropylene macroporous meshes fixed with surgical glue or polypropylene suture.

## METHOD

The project was submitted and approved by the Ethics Committee on Animal Use (CEUA) of the State University of Ponta Grossa (no 0033168, July 5, 2019), according to 11.794 Federal law (October 8, 2008), which regulates the scientific procedures with animals, and the Brazilian Academy of Animal Experiments (COBEA).

A total of 20 Wistar rats, males, with 3 months, weighing 280-300 g from the Central Biotério of the State University of Ponta Grossa, were used. The animals were divided into 2 subgroups of 10: (1) surgical glue subgroup C1, n=10, in which macroporous polypropylene mesh was fixed using surgical adhesive and (2) suture subgroup C2, n=10, in which polypropylene 5.0 thread was used to fix the mesh in extraperitoneal position. Each animal of the subgroups was designed by a number from 1 to 10.

Both groups were submitted to similar surgical procedures, a lightweight, monofilament polypropylene mesh, with an estimated weight of 44 g/m², being macroporous, and with a dimension of approximately 6.29 mm².

### Surgical mesh

**Figure 1 -:**
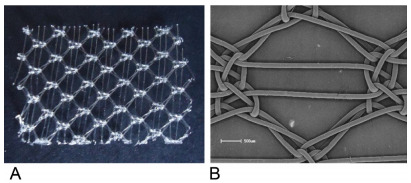
(A) Bard Soft^®^; (B) Bard Soft^®^ (Scanning electron microscope).

It was used a Bard Soft mesh^®^ (Figure 1).

The rats underwent preoperative fasting of 12 h and were anesthetized with atropine sulfate (0.05 mg/kg body weight) intraperitoneally and, after 10 min, the mixture of 2% xylazine hydrochloride (10 mg/kg) and hydrochloride of ketamine 10% (25 mg/kg). When necessary, half the dose was repeated after 20-30 min. They were submitted to postoperative analgesia with oral acetaminophen in the dose of 40 drops for every 500 ml of water offered in the first 2 days. Euthanasia was performed on the 90th postoperative day. At that time, a macroscopic evaluation of the operative wound and the peritoneal cavity was made.

### Surgical procedure

**Figure 2 -:**
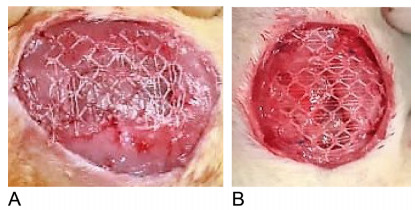
Meshes applied to the abdominal wall defect: (A) Surgical glue and (B) suture.

A defect of 1x2 cm was produced in the abdominal wall, preserving the integrity of the parietal peritoneum. The correction was performed using each of the 1.5x2.5 cm area meshes fixed in the extraperitoneal position with four drops of surgical glue (2-octyl cyanoacrylate) in mesh angles in the C1 subgroup (Figure 2A) and four separate stitches of Prolene^®^ 5-0 wire securing the mesh angles to the aponeurosis of the abdominal wall, 0.5 cm from the edge of the lesion, in the C2 subgroup (Figure 2B). The skin was sutured with intradermal 5-0 mononylon intradermal.

### Euthanasia and material collection

Euthanasia was performed at the 90^th^ postoperative, with an intraperitoneal injection of an overdose of ketamine and xylazine.

**Figure 3 -:**
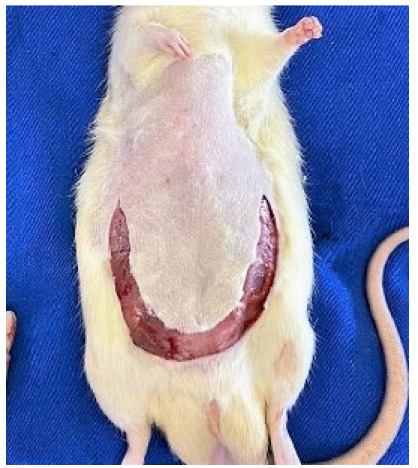
U-shaped incision.

A U-shaped caudal incision was made in the skin, subcutaneous cellular tissue, aponeurosis, and abdominal musculature, which started at the coastal edges and outlined the suprapubic region, with the exposition of abdominal cavity (Figure 3). At that time, the macroscopic evaluation of the operative wound and the peritoneal cavity was made, as was observed the mesh condition and also whether hematomas, infection, or adherences were present.

**Figure 4 -:**
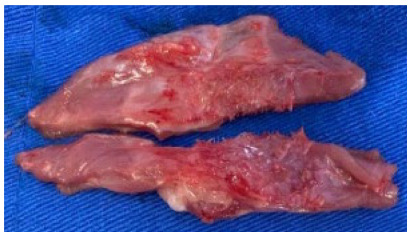
Cranial and caudal fragments of abdominal wall.

The segments of the abdominal wall were divided with a median cut and resulted in cranial and caudal fragments (Figure 4). The cranial segments containing the mesh, aponeurosis, musculature, and peritoneum were maintained in cold isotonic saline and kept in vials with ice. On the same day, they were submitted to tensiometric tests. The caudal fragments were maintained in 10% formalin solution. The cranial segments were placed in vials with isotonic saline solution and kept in vials with ice and were submitted to microscopic analysis. For tensiometry, the Shimadzu (Japan) model AG-I tensiometer was used with Trapezium 2 software, where the data provided for the test (area and thickness of the tissue) and the results obtained were recorded. The tests were performed at a temperature of 24°C. The apparatus was calibrated for a speed of 50 mm/min. The results were expressed as Newton (N). The cranial fragment was attached to the tensiometer by the muscular tissues next to the suture site.

### Microscopic analysis

The table of Vizzotto et al. ^24^ was used for the quantitative analysis of inflammatory parameters.

The pieces were cut by a microtome each 5 μm thick. The slides were stained with hematoxylin and eosin, Masson’s trichrome, and picrosirius red in order to analyze types I and III^13^ collagen and were evaluated by a pathologist who had no prior knowledge of the animal group. In each slide, it was evaluated the presence of foreign body granuloma, inflammatory response, and fibrosis.

The inflammatory parameters were submitted to quantitative analysis, according to the Vizzoto et al.^24^ methodology. The study focused on the microscopic field and was characterized by the observation of neutrophils, edema, congestion, monomorphonuclear, granulation tissue, and fibrosis. The data were classified as accentuated (3), moderate (2), mild (1), and absent (0), according to the intensity.

The images for types I and III collagen were obtained using Olympus AX70 microscope - with the attached polarizer. The settings of the camera and light intensity were the same in each sample, in order to avoid the variations related to the capture process. The images were analyzed using the Photoshop CS6 program to contrast colors and ImageJ to quantify pixels. The results were expressed in the percentage of fibers’ types I and III.

### Statistical analysis

To analyze the tensiometric variance, non-parametric statistic tests were used to evaluate the inflammatory process of different materials and fibrosis. Kolmogorov-Smirnov (KS) normality test and Student’s t-test were processed in order to compare the results. The level of rejection of the null hypothesis was 0.05% or 5%.

## RESULTS

### Tensiometric evaluation

The tests were performed at an ambient temperature of 24°C. The equipment was calibrated for a speed of 50 mm/min, and the results were expressed in Newton (N). Each cranial fragment was measured using a pachymeter, and it was attached to the tensiometer by the muscular tissues next to the suture site.

In tissue tensiometry with implanted tissue, it was found that the rupture always occurred outside the suture line of the mesh in the abdominal wall.

The mean stress at break of the subgroup C1 (surgical glue) at 90 postoperative days was 28.47±11.39 N; it did not present a statistically significant difference in relation to the subgroup C2 (suture) with mean tension of 32.1±10.59N (p=0.4706).

### Macroscopic evaluation

No animal presented hematoma, infection, fistula, suture dehiscence, or incisional hernia, and the edges of the mesh fixation to wounds were fully coated in all animals.

### Microscopy

**Figure 5 -:**
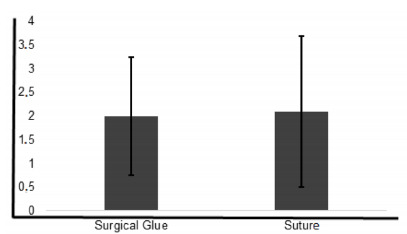
Mean and standard deviation of inflammatory phase.

The final score of the inflammatory process characterization revealed that both subgroups were in the subacute phase. There was no statistical significance when subgroups were compared (p=0.380, Figure 5).

### Masson’s trichrome

**Figure 6 -:**
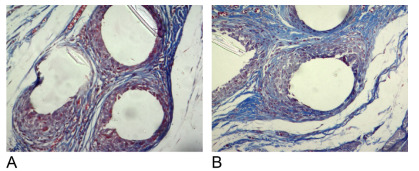
(A) Surgical glue and (B) suture (20X).

Filamentous encapsulation is observed when surgical glue and polypropylene suture are used, which shows its importance in the integration of the mesh into receptor tissues and also provides greater flexibility to the wall after incorporation (Figures 5 and 6).

### Picrosirius red

On the 90th postoperative day, types I and III collagen analysis did not show a statistically significant difference between the subgroups (type I - p=0.3234 and type III - p=0.0703).

## DISCUSSION

The progress of surgical mesh fixation techniques has improved the herniorrhaphy procedure^5^ ^,^ ^8^ ^,^ ^11^. The possibility to use surgical mesh showed satisfactory results in reducing postoperative chronic pain^6^ ^,^ ^10^ ^,^ ^17^, and it is not related to higher recourrence^12^ ^,^ ^15^ ^,^ ^18^ ^,^ ^20^. Several studies have listed the advantages of surgical glue compared to sutures, such as lower tissue aggressions, lower adherences grade, and also better local acceptance^13^ ^,^ ^15^.

The polypropylene meshes chosen for this study were macroporous, monofilament, and with lightweight. The broad pores show less inflammatory infiltrate and greater incorporation of mesh into the tissue^2^ ^,^ ^25^.

In tissue tensiometry, rupture was observed always outside the suture line, a result also obtained by Utrabo et al.^21^ It means that the fixation either with four stitches or four drops of surgical glue the mesh angles provided sufficient incorporation.

Dilege et al.^5^ in an experimental study with rats comparing the mesh fixation *n*-butyl-cyanoacrylate or polypropylene suture did not show a statistical difference in terms of adhesion and rupture tension when groups were evaluated at 21th and 42th postoperative days. Inflammatory response, fibrosis, and tissue growth were also equivalent. However, it was demonstrated that glue fixation results in less foreign body reaction.

Another similarly outlined study^20^ did not demonstrate macroscopic differences after mesh application with suture or fibrin glue to repair the preperitoneal defect in rats. The tensiometric tests to which these samples were submitted have also revealed similarities between the tested groups. Moreover, both fixation methods exhibited the same cellularity, except close to polypropylene suture, where there was greater macrophage infiltration.

Schreinemacher et al.^17^ have investigated the adhesions at the 7th and 90th postoperative days after disposing of surgical mesh in the intraperitoneal position. Absorbable fixation methods, such as fibrin glue, and non-absorbable techniques, such as polypropylene sutures, were compared. In this case, it was observed that glue has not caused adhesions and also had a limited inflammatory response. Between non-absorbable methods, a fibrosis capsule was observed around the correction site, which did not occur in absorbable methods.

The results of this study, in the comparison of the mean of rupture tension between the C1 (surgical glue) and C2 (suture) on day 90, did not show superiority to fine method. This fact was also reported by the studies previously mentioned^5^ ^,^ ^20^.

The results show that despite the fixation technique prosthesis resistance is adequate. Insufficient tension values may indicate that the material does not have elasticity and may result in hernia reoccurrence, prolapse, or pain^25^.

The evaluation of inflammation by the score according to histological findings of C1 and C2 subgroups has also shown equal inflammatory process at the 90th day after prosthesis fixation. The subacute inflammatory phase was prevalent after this period, and the acute inflammatory stage was not found.

Therefore, it is observed that both techniques have the same tissue growth, inflammatory infiltration, and fibrosis^5^.

Zhu et al.^25^ reported that mesh incorporation attracts inflammatory cells, such as macrophages, and it initiates a response against the foreign body. This reaction has to be balanced to result in adequate tissue replication and provides biocompatibility and good clinical performance.

Hollinsky et al.^8^ showed a gradual decrease in the inflammatory process after 2 months of prosthesis implant. At this stage, only a few inflammatory cells are found around the mesh, which proves better incorporation with tissue during the weeks.

Studies^2^ ^,^ ^5^ ^,^ ^17^ ^,^ ^20^ ensure that surgical glue is an effective alternative to suture, with no influence in the inflammatory process of material incorporation. Both methods are safe and can provide appropriate tension force to an ideal overlap of mesh and abdominal wall. The inflammatory response has also been satisfactory between groups, which ensures proper healing from prosthesis and organisms interaction.

The results of this study did not show a significant difference in collagen proportion between groups. Type III collagen predominates in initial healing and type I collagen is found in a greater amount at late healing, after the remodeling phase^24^.

According to the study by Sofii et al.^19^, delays in defect healing can occur when type III collagen retards to decrease and type I collagen takes a longer time to spread. It was not observed in the present study.

## CONCLUSION

Surgical glue or suture can be used to fix the macroporous polypropylene mesh to repair the abdominal wall defect. Both methods are equally effective.
